# What was retained? The assessment of the training for the peer trainers' course on short and long term basis

**DOI:** 10.1186/1471-2458-8-24

**Published:** 2008-01-22

**Authors:** Vildan Mevsim, Dilek Guldal, Nilgun Ozcakar, Ozge Saygin

**Affiliations:** 1 Department of Family Medicine, Dokuz Eylul University Medical Faculty, Izmir, Turkey; 2 Department of Econometrics, Dokuz Eylul University Faculty of Economics and Administrative Sciences, Izmir, Turkey

## Abstract

**Background:**

In Turkey, the studies have reported that the age at which sexual intercourse and sexual activity starts has been steadily declining. There is an urgent need to increase social and health services for young people in order to provide them with a healthy life by changing their risky behaviors, avoiding unwanted pregnancies and sexually transmitted diseases (STDs). Sexual and reproductive health training particularly for adolescents warrants special attention and consideration.

The objective of our study is to find out the short and long term effectiveness of a training course on peer education.

**Methods:**

The study was conducted on 237 students who participated in a 40 hour Peer Trainer Training course. We utilized two types of evaluation methods to measure the effectiveness of the training on students' knowledge and attitude. The first method consisted of administering 3 tests comprised of the same 45 questions at 3 separate time intervals. Prior to the training a pre-test was given to obtain a measurement of base knowledge, and then an immediate post-test was given to evaluate the change in the knowledge and opinion of the participants.

Finally, 6 months later the same test was administered to measure the retention of knowledge by the students. In the second type of evaluation, the participants' assessment of the training itself was sought by asking them to complete a Short Course Evaluation Form. We utilized SPSS 12.0 for descriptive analysis, and the Wilcoxon two related sample t-test were run.

**Results:**

According to the pre and immediate post-test results, the training resulted in an increase in knowledge learned by an average of 21.6% (p < 0.05). Whereas, according to the immediate post test and the late post-test which was given six month later, there was a 1.8% decrease in the knowledge and attitude of the participants (p > 0.05). Participants thought that they had fun during training, and they became aware of what they knew and what they did not know.

**Conclusion:**

Peer trainers with the training methods utilized, the knowledge and counseling acquired during training sessions will be able to provide counseling to their peers on reproductive health.

## Background

The population of Turkey continues to get younger while the percentage of the population that is capable of reproduction is rising with an expected increase of 40% by 2025. Furthermore, studies have reported that the age at which sexual intercourse and sexual activity starts has been steadily declining. There is an urgent need to increase social and health services for young people in order to provide them with a healthy life by changing their risky behaviors, avoiding unwanted pregnancies and sexually transmitted diseases (STDs). Since the appearance of HIV/AIDS, sexual health issues have gained more importance and require that they be handled with more care [1].

Sexual and reproductive health training particularly for adolescents warrants special attention and consideration. Studies have verified that the current young generation, including university students in Turkey are not well informed about sexual health making it difficult for them to engage in healthy sexual behavior and display a healthy attitude [2-5].

Adolescents commonly learn about sex through their friends and peer groups [6,7]. Taking this fact into consideration, peer training would be one of the most effective methods used for training adolescents on sexual and reproductive health [7-12]. As it is generally known, "Peer Education" has been described as the development of knowledge and skills by means of providing effective help and support among those who are of the same status [13].

Peers should be given special training so that they are able to provide counseling and training services on sexual and reproductive health [7-12,14]. Many studies have shown that most knowledge learned is soon forgotten after its acquisition, if that knowledge is not applied.

In this study our objective was to find out the short and long term effectiveness of a training course on peer education.

### Project

The training of peer educators has taken place as an activity of the "Modern Stork Legends" project within the scope of Turkey Reproductive Health Program supported by the European Union. The aim of this project is to contribute to the creation of an educated and aware society and a healthy generation by increasing the information available, changing attitudes and behaviors on sexual and reproduction health of the youth, who live in the Buca-Izmir region and are aged from 16–25.

To achieve this goal, a wide array of activities was organized in 2006. The major activity was peer education.

## Method

The study was conducted at Dokuz Eylul University in Izmir. The institutional review board of the university approved the research. According to the project goals, 11 training sessions were planned to train peer trainers. 255 students from Dokuz Eylul University responded to the training session notices which were placed on the internet. During the interviews, applicants were told that they were expected to share what they had learned afterwards with their peers. They were also informed that teaching would be done on a one-to-one basis instead of teaching in small groups because of the sensitivity of the topic. They were instructed to give information whenever and wherever it was demanded and carried to environments where students felt comfortable themselves (informal places such as canteens, classrooms, library, or during leisure activities). Peer educators had to take notes for every interview in a separate file, and then review them monthly with their supervisor. The students also were told that the project was completely voluntary and no fees would be paid for their time. After the initial interviews, 237 students agreed to participate in the training. 11 training sessions of peer trainers took place with an average of 21.54 (min = 18 max = 27) students in each course. All 237 students attended the course and all completed the course. All training sessions were given by four trainers who were medical doctors and nurses and had participated in a training of trainer's course.

Each course was structured as a total 40-hour program. The training curriculum consisted of three main topics, which were reproductive health, peer education and counseling. Various active learning methods were utilized to teach these three topics (Table 1). At the end of the course, the graduates were informed that they would be paid five Euros for each interview they obtained as compensation for expenses that they might have incurred (such as treating friends, having some snacks together with the trainees or making some photocopies etc.). The reimbursement for expenses, however, could never exceed 30 Euros per month regardless of the number of interviews. The payments were made based on the evaluation of their files.

**Table 1:** The program of training of peer trainers

**Topic**	**Methodology**	**Duration**
**FIRST DAY**		
Project presentation	Presentation	30 minutes
What is reproductive health?	LGD*, Group brainstorming	15 minutes
What is reproductive health I	Presentation	30 minutes
Reproductive rights	SGD** and debate	30 minutes
What is reproductive health II	Presentation	20 minutes
What is healthy sexual life?	SGD and case studies	1 hour
Healthy sexual life and respect for different sexual orientation	Presentation	30 minutes
**SECOND DAY**		
Who is adolescent?	Presentation	30 minutes
Communication with a peer	SGD and role-play	30 minutes
Communication	Presentation	30 minutes
Peer communication	LGD and SGD	30 minutes
What is peer, what is peer education	Interactive Presentation	30 minutes
Needs assessment for learning process and counseling	Interactive Presentation	30 minutes
**THIRD DAY**		
Reproductive physiology and anatomy	Presentation	30 minutes
Reproductive physiology and anatomy	Demonstration on models	30 minutes
What is family planning and the challenges	Presentation	30 minutes
Methods for family planning	SGD	30 minutes
Methods for family planning I	Presentation and demo.	30 minutes
Methods for family planning II	Presentation and demo.	30 minutes
Methods for family planning III	Presentation and demo.	30 minutes
**FOURTH DAY**		
What is STDS***? AIDS and Hepatitis	Presentation	30 minutes
STDS*-Risky behaviors and situations	LGD	30 minutes
Other STDS*	Presentation	20 minutes
Substance abuse and addiction	Presentation	30 minutes
Peer education practice I	Role-Play	1 hour
Peer education practice II	Role-Play	1 hour
Prevalent reproductive problems	Presentation	30 minutes
Peer education practice I	Role-Play	1 hour
Peer education practice II	Role-Play	1 hour
What next? Tools for monitoring the peer counseling		1 hour

*LGD: Large group discussion

**SGD: Small group discussion

***STDs: Sexually transmitted diseases

During the project period, peer educators were engaged 6800 counseling sessions in total.

The efficacy of the training was measured by two methods. In the first, a pre-test with 45 questions was applied to evaluate the base knowledge and opinion of the participants. True and false types of test questions were prepared meeting the objectives of the course, making sure that they covered all training topics. The same test was applied later twice, one immediately at the conclusion of the course, the other six months later. The pre and immediate post test was given to all contributing students but the post test, was given to only 92 students who were chosen at random. The reason for selecting this option was that 38 students decided not to further participate in the project and did not provide training to their peers. Therefore, these 38 students did not use the knowledge received during the courses and may have forgotten a lot of it. Although 41 participants had already graduated, they were still included in the randomization. In addition, six months after the final course, the summer holiday had started and many students had gone out of town for vacation or returned to their home towns in different cities which made it difficult to get in touch with them. So we decided to study a smaller representative group instead of the entire group of participants. According to calculations made by EpiInfo 6, taking 70% as true pretest score and 90% as true post test score with 95% CI and 95% power, 111 students were chosen at random. In this study, rate of change was expected as a high value. Because the high motivation of the students who apply to the reproductive health training, their being volunteers, their getting knowledge by active learning and their being highly motivated and also their being university students and the interesting content of education kept expectations of change high.

We contacted them through the internet and invited them to the main office. 92 students came and filled out the same test that had been used for the pre and immediate post test. The power calculated with 92 participants was 90%.

In the second type of evaluation, the opinion of the participants on the training was obtained. In this evaluation, which was completed at the end of the course, the Short Course Evaluation Form was filled out by the participants. In this form, the evaluation of the course had been completed using a likert scale over 15 parameters.

SPSS 12.0 and EpiInfo 6 was used for statistical analysis. The effectiveness of the training was measured by taking into account the ratio of correct answers in the pre-test, immediate post-test, late post-test. To compare the results of pre-test with immediate post-test, and immediate post-test with late post-test, the Wilcoxon two related sample t-test was run. In the evaluation of the training, participation rates were evaluated.

## Results

Distribution of participating students on gender, age and school is indicated in Table 2. The age average is 21.18.

**Table 2:** Distribution of participants according to their demographic variables

	Number	Percentage
Gender		
Female	127	53.59
Male	110	46.41
		
Age		
≤19 years old	27	11.39
20 years old	47	19.83
21 years old	75	31.65
22 years old	52	21.94
23 years old	26	10.97
≥24 years old	10	4.22
		
School attended		
Faculty of Education	102	43.04
Faculty of Economics and Administrative Sciences	57	24.05
Faculty of Engineering	20	8.44
Faculty of Medicine	15	6.33
Faculty of Arts and Sciences	14	5.90
Faculty of Business	9	3.80
School of Physical Therapy and Rehabilitation	8	3.38
Vocational School	3	1.27
Faculty of Fine Arts	2	0.84
School of Nursing	2	0.84
School of Maritime Business And Management	2	0.84
The Institute of Health Sciences	2	0.84
School of Foreign Languages	1	0.42
		
TOTAL	237	100.00

### Knowledge and opinion changes of the participants

The average number of correct answers in the pre-test in the 11 groups trained was 69.7% while the average in the immediate post-test was 91.3% and the difference between the two rates was statistically significant (p < 0.05). The changes in knowledge according to course groups have been shown in the Table 3.

**Table 3:** The mean percentage of success increase and the mean percentage of accurate answers in the pre-and immediate post-test of the training groups

**GROUP**	MPAA** in Pre-test (%) ± SD	MPAA** in Immediate Post-test (%) ± SD	**Percentage Increase (%)**
**A**	73.1 ± 10.77	92.4 ± 3.97	19.3*
**B**	72.0 ± 10.69	92.0 ± 3.41	20.0*
**C**	69.6 ± 11.95	93.6 ± 3.13	24.0*
**D**	70.0 ± 8.53	90.9 ± 3.57	20.9*
**E**	67.6 ± 12.87	91.8 ± 1.86	24.2*
**F**	71.1 ± 11.70	90.7 ± 4.63	19.6*
**G**	68.4 ± 10.33	90.2 ± 4.64	21.8*
**H**	69.3 ± 7.55	90.7 ± 3.58	21.3*
**I**	68.9 ± 11.83	88.9 ± 4.71	20.0*
**J**	69.8 ± 9.86	90.2 ± 3.66	20.4*
**K**	66.4 ± 10.37	92.4 ± 4.17	26.0*
**TOTAL**	69.7 ± 10.56	91.3 ± 3.99	21.6*

*p = 0.00

**MPAA: Mean percentage of accurate answers

In the late post-test which was administered after 6 months, the average correct answer was 89.4% and the knowledge level decreased by 1.8% (p > 0.05).

Table 4 shows the changes in knowledge and opinions according to the question categories. The questions were divided into 10 sections according to question topics. When the change in knowledge was examined at the groups' levels, the most significant knowledge change was observed in counseling and family planning. Table 4 also shows the changes of mean percentages among the pre and the two post tests according to question categories. The reliability of the test for the total score for each of the three time periods was medium and low (cronbach alpha of pre-test: 0.759, cronbach alpha of immediate post-test: 0.168 and cronbach alpha of late post-test:0.211).

**Table 4:** The mean percentage of success change according to the categories of questions and the statistical significance of changes

**Category of Questions**	MPAA** in Pre-test (%) ± SD	MPAA** in Immediate Post-test (%) ± SD	MPAA** in Late Post-test (%) ± SD	**Change in Percentage of Pre and Immediate post-test (%)**	**Change in Percentage of Immediate and Late post-test (%)**
**Counseling**	48.20 ± 40.29	99.50 ± 0.30	84.77 ± 13.05	51,30*	-14.73^§^
**Family planning**	61.16 ± 27.19	98.58 ± 0.88	96.34 ± 3.85	37.42*	-2.24^§^
**Reproductive physiology**	61.55 ± 29.94	83.05 ± 27.07	82.08 ± 22.35	21.05*	-0.97^§^
**Sexually transmitted diseases**	79.98 ± 13.50	98.06 ± 1.65	95.64 ± 3.67	18.08*	-2.42^§^
**Respect for different sexual orientation**	76.23 ± 12.28	95.57 ± 4.02	94.58 ± 2.93	19.34*	-0.99^§^
**Peer education**	77.32 ± 18.08	96.30 ± 9.87	93.08 ± 9.63	18.97*	-3.22^§^
**Substance abuse and addiction**	76.43 ± 4.52	93.01 ± 3.63	86.60 ± 11.50	16.58*	-6.41^§^
**Concept of reproductive health**	60.74 ± 34.97	74.76 ± 40.23	75.64 ± 35.71	14.02*	+0.88^§^
**Adolescence**	87.20 ± 11.70	97.17 ± 2.5	96.03 ± 3.81	9.97*	-1.14^§^
**Communication**	80.42 ± 12.71	87.88 ± 13.13	84.78 ± 18.19	7.46*	-2.10^§^
**Total**	69.7 ± 10.56	91.39 ± 17.86	89.10 ± 16.56	21.6*	-2.29^§^

* p < 0.05

**MPAA: Mean percentage of accurate answers

^§ ^p > 0.05

Keeping in mind that overall score is not reliable but there are significant and large differences between the pre test and the post test. Due to the fact that these overall scores are not reliable, the usage of these results as a scale for other studies is not advisable.

### Participants' evaluation of the training

When all training classes came to an end, the participants were asked to fill out a form to evaluate the training. Table 5 shows the answers in percentages (Table 5). The reliability of the evaluation scale utilized is found to be cronbach alpha = 0.865

**Table 5:** The participants' assessment of the training itself

**QUESTIONS**	**Agree (%)**	**Not Agree (%)**	**No Idea (%)**
All I want to learn about the basics of the course have been covered.	95.7	3.0	1.3
It was worth the time I spent fort he course.	97.4	0.4	2.2
Participants would be involved in the course rapidly.	95.2	1.3	3.5
My expectations were appreciated.	98.7	0.9	0.4
Some of my ideas and opinions have changed because I joined in the course.	75.7	19.6	4.7
We had enough time to sink in the information delivered to us.	67.8	20.0	12.2
I felt being supported.	95.2	1.7	3.1
I was enlightened about what I know.	99.1	0.9	0.0
I was enlightened about what I need to know.	98.7	0.9	0.4
The course will facilitate behavioral change.	92.2	3.5	4.3
I want to learn more on this topic after the course ends.	85.7	7.0	7.3
I had a good time during the course.	100.0	0.0	0.0
Facilitators did a good job getting the participants included.	98.3	0.0	1.7
Facilitators had conflict resolution skills.	92.2	0.0	7.8
Facilitators helped reaching the objectives of the course.	92.2	0.0	7.8

## Discussion

The results of the pre and the immediate post-tests indicate that the training delivered had a positive impact on the level of knowledge of the participating students as it had in other studies [15,16]. Unfortunately, in the Turkish education system courses on sexual and reproductive health have only been recently integrated into the university curriculum. Most of these courses are restricted to biology and physiology. Turkish adolescents learn about sexual and reproductive health mostly through media [6]. This was the main reason why there was an expectation for students to have some information on this topic.

In the pre-test evaluation, it was discovered that students had more knowledge of the traits of adolescence, communication and sexually transmitted diseases. There may be a couple of reasons for this. Adolescents learn the traits of adolescence and communications as part of their daily life experience. In addition, their knowledge of sexually transmitted diseases, particularly AIDS and Hepatitis B, might be due to the very keen educational programs of the Ministry of Health and International Organizations in recent years [11].

According to the pre-test results, students had the lowest level of knowledge in counseling, reproductive health, the concept of sexual and reproductive health, reproductive physiology and anatomy, and family planning. The fact that students do not know much about family planning implies that university students are at risk of unwanted pregnancies. We concluded that students closed this knowledge gap after training. The increase in their level of knowledge in counseling was important as the students would engage in peer counseling.

The most significant problem in education is retaining knowledge that is not actively used. Some studies report a knowledge loss from 8.6% to 46.5% [18,17]. Despite the 1.8% drop, the knowledge level was still high after 6 months. We believe this is due to peer educators using their newly acquired knowledge in their personal lives, and transferring the knowledge to their peers. The only drop in knowledge level was on substance abuse. The reason for that also may be due to the fact that they may never have or rarely used the knowledge on substance abuse during the 6 months. There are strict laws and enforcement of narcotic substances making their use less common.

The educational techniques that we used, such as group discussions, role-plays and demonstrations might have helped to increase and reinforce and retain the knowledge students had received, especially on family planning and counseling [15,16,19-21].

Female students had a special interest in the training, which was important as we were expecting a low level of interest due to the assumed unacceptability or unwillingness to participate in such training.

At the end of the course, students were told to fill out an evaluation form to measure the efficiency of the training. The students expressed that they enjoyed the training and had a good time. Even though training generally took place over the weekends, on the whole regular attendance was maintained and students showed great interest. We think the educational techniques and methods utilized had a role in that. The most important feature of adult education is to have an enabling environment where the participants are able to express themselves and exchange experiences [22]. The ability to distinguish what they know from what they do not know on sexual and reproductive health will help them protect themselves and inform people around them.

The participants said that some of their opinions did not change, and that they needed more time to process the new information. The change in knowledge, and its effect on attitude and behavior change of the students will be further evaluated at later stages of the project. Attitude and behavior change would occur in the long run. Due to cultural and traditional reasons, it would take a longer time and more effort to change some attitudes and beliefs. Therefore, we will need to conduct more training, set up institutions where youth can receive regular counseling services.

## Conclusion

Providing training by using active learning methods for counseling and reproductive health may help create young trainers who train their peers and keep their knowledge fresh by teaching others. Not only students, but society as a whole will benefit from this training through peer counseling.

## Competing interests

The author(s) declare that they have no competing interest.

## Authors' contributions

VM in the conception and design, writing of draft manuscript

DG revising critically, and given final approval

NO acquisition of data

OS Analysis and interpretation of data

All authors read and approved the final manuscript.

## Pre-publication history

The pre-publication history for this paper can be accessed here:


